# Metformin-Induced Hemolysis in a Glucose-6-Phosphate Dehydrogenase-Deficient Patient: A Case Report

**DOI:** 10.7759/cureus.65081

**Published:** 2024-07-22

**Authors:** Adil Jumani, Hadiza Ibrahim, Hana Purra, Alaa K Alkhazraji, Majdi S AlNajjar

**Affiliations:** 1 Internal Medicine, Zayed Military Hospital, Abu Dhabi, ARE; 2 Endocrinology and Diabetes, Zayed Military Hospital, Abu Dhabi, ARE

**Keywords:** metformin, hemolytic crisis, drug-induced hemolysis, diabetes mellitus (t2dm), glucose-6-phosphate-dehydrogenase deficiency (g6pd)

## Abstract

Metformin is a first-line medication used in the treatment of type 2 diabetes mellitus along with other conditions such as insulin resistance and polycystic ovarian syndrome. Overall, metformin appears to be well tolerated with a low incidence of side effects; however, in certain high-risk populations, it can trigger a hemolytic crisis. This case report describes a middle-aged man who was initiated on metformin for new-onset diabetes, following which he had an acute hemoglobin drop and was diagnosed to be having a hemolytic crisis requiring hospitalization. He was diagnosed with a deficiency of the enzyme glucose-6-phosphate dehydrogenase (G6PD) on admission. Extensive workup was done to rule out other causes of hemolysis, all of which came back to be negative. The offending agent was stopped and the patient received supportive care after which he improved. This case highlights a rare, yet important, side effect of metformin that needs to be observed in certain individuals, especially patients with G6PD deficiency. Routine testing of high-risk populations known to be G6PD deficient should be considered before initiating metformin.

## Introduction

Metformin is among the first-line oral anti-diabetic agents used to manage pre-diabetes and type 2 diabetes mellitus. Its spectrum of adverse effects ranges from trivial gastrointestinal upset to severe conditions such as lactic acidosis and bradyarrhythmias [1]. Glucose-6-phosphate dehydrogenase (G6PD) enzyme deficiency is an X-linked recessive disorder and the most common erythrocyte enzyme disorder, affecting approximately 500 million people worldwide. Deficiency of this enzyme leads to red blood cell breakdown, resulting in hemolytic anemia [2]. While many foods and drugs are known triggers of hemolysis in G6PD-deficient patients, metformin is rarely reported as a cause [3]. We present a case of a young Middle Eastern man who developed severe hemolytic anemia within two weeks of starting metformin for newly diagnosed diabetes.

Metformin, a first-line treatment for type 2 diabetes, is generally well-tolerated but associated with several adverse effects. The most common are gastrointestinal issues like nausea, vomiting, and diarrhea, affecting up to 20% of patients [4,5]. These symptoms are typically mild and manageable. Rare but severe adverse events include lactic acidosis, which occurs predominantly in patients with underlying conditions such as renal or hepatic impairment [4,5]. Despite these risks, the long-term safety profile of metformin is favorable, with research indicating it does not significantly increase the risk of major adverse cardiovascular events (MACEs) compared to other diabetes medications [4].

Metformin-induced hemolysis is an extremely rare adverse event, especially in patients with G6PD deficiency [6-11]. G6PD deficiency is an X-linked recessive disorder affecting approximately 500 million people worldwide and leads to hemolytic anemia when red blood cells are exposed to oxidative stress [2]. In such patients, metformin can exacerbate oxidative stress, leading to the destruction of red blood cells and subsequent hemolysis.

Management of metformin-induced hemolysis involves the immediate discontinuation of metformin and providing supportive care, including hydration and monitoring of hemoglobin levels. In severe cases, blood transfusions may be necessary. It is also crucial to identify and avoid other potential triggers of hemolysis in G6PD-deficient patients to prevent recurrent episodes [2].

Several drugs are known to trigger hemolysis in patients with G6PD deficiency. These include certain antimalarials like primaquine and chloroquine, sulfa drugs such as sulfamethoxazole and sulfadiazine, analgesics like high-dose aspirin and phenazopyridine, and antibiotics, including nitrofurantoin, dapsone, and ciprofloxacin. Additionally, some antidiabetic drugs, such as glibenclamide, can also cause hemolysis [12]. Patients with G6PD deficiency should be carefully screened for these medications and counseled on avoiding them to minimize the risk of hemolytic episodes.

## Case presentation

A 40-year-old previously healthy male presented to the emergency department with a two-day history of tiredness and fatigue. He stated that until two days ago, he had been fine and able to run miles; however, during the last two days, he has experienced severe tiredness and exertional dyspnea with minimal exertion. He also complained of chest tightness, palpitations, and associated dizziness. Since the onset of symptoms, he has noticed jaundice over his entire body. He had one episode of low-grade fever the day before presenting to the emergency room but reported no respiratory symptoms. His urine appeared dark orange, although he denied any abdominal pain or changes in bowel habits. He also denied having any rashes or bleeding from other sites and reported no recent illnesses or contact with sick individuals.

His past medical history was significant for type 2 diabetes mellitus, diagnosed two weeks before presentation. He was started on alogliptin 12.5 mg twice daily, metformin 1000 mg twice daily, empagliflozin 10 mg daily, and methylcobalamin 500 mcg daily. He reported intentional weight loss of 12 kg over three months through diet and exercise, with no personal or family history of blood disorders. He had recently traveled to Switzerland and Egypt but denied any tick or insect bites and reported no ingestion of fava beans. He admitted to being an active smoker.

On physical examination, including a per rectum examination, he was normal apart from generalized skin jaundice and scleral icterus. He was hemodynamically stable, with a heart rate of 110 beats per minute and a temperature of 38°C.

Laboratory investigations were done in the emergency room and were as follows when compared to his results two weeks ago (Table 1).

**Table 1:** Laboratory values

Test	On admission	2 weeks ago	Reference values
White blood cells	11.29 x 10^3 ^cells/uL	4.5 x 10^3 ^cells/uL	4.5 – 11 x 10^3 ^cells/uL
Hemoglobin	5.3 g/dL	15.3 g/dL	13 – 18 g/dL
Mean corpuscular volume (MCV)	83.3 fl	85 fl	80 – 100 fl
Mean corpuscular hemoglobin (MCH)	28 pg	29 pg	27 – 32 pg
Mean corpuscular hemoglobin concentration (MCHC)	32.4 g/dL	33 g/dL	31 – 36 g/dL
Platelets	253 x 10^3 ^cells/uL	247 x 10^3 ^cells/uL	150 – 450 x 10^3 ^cells/uL
Reticulocytes	0.3%	0	0.5 – 2.0 %
International normalized ratio (INR)	1.03	-	1.0 – 1.1
Total bilirubin	69.6 micmol/L	9.8 micmol/L	0 – 20 micmol/L
Direct bilirubin	13.5 micmol/L	4.5 micmol/L	0 – 5 micmol/L
Lactate dehydrogenase	552 unit/L	-	125 – 220 unit/L
Iron	39 micmol/L	-	11 – 28 micmol/L
Total iron binding capacity	39 micmol/L	-	45 – 80 micmol/L
Iron saturation	100%	-	20 – 50 %
Ferritin	5667 micg/L	-	20 – 275 micg/L
Folate	25.4 nmol/L	-	7 – 46 nmol/L
Vitamin B12	349 pmol/L	-	138 – 652 pmol/L
Glycosylated hemoglobin (HbA1c)	6.1%	12.1%	>/= 6.5%
Direct antiglobulin test (DAT)	Negative	-	Negative

A peripheral smear was performed upon admission, which revealed normocytic normochromic anemia with anisocytosis, without evidence of schistocytes or bite cells. Hemoglobin electrophoresis yielded normal results. A G6PD screen showed G6PD enzyme deficiency.

He was diagnosed with an acute hemolytic crisis likely secondary to medications, primarily metformin. Upon admission, he received a blood transfusion consisting of two units of packed red cells, which increased his hemoglobin level to 8.2. Following discharge, he was evaluated by an endocrinologist, who initiated treatment with sitagliptin, pioglitazone, and empagliflozin for his diabetes.

Two months after discharge, his follow-up blood tests at the clinic revealed a hemoglobin level of 14.3. A repeat G6PD screen confirmed the diagnosis of G6PD deficiency.

## Discussion

Drug-induced hemolytic anemia is a rare occurrence. While metformin is generally considered safe in patients with G6PD deficiency, there are numerous reports of it causing a hemolytic crisis in such individuals [6]. Associations have been noted in pediatric cases presenting with hemolytic anemia during diabetic ketoacidosis episodes, whereas, in adults, it typically manifests during initial treatment phases [7].

Despite our patient being on several medications, metformin was identified as the likely cause, as cessation of the drug resulted in symptom resolution. He did not exhibit other triggers of G6PD hemolysis such as bacterial or viral infections, ingestion of fava beans, or drugs known to induce hemolysis like antibiotics or antimalarials [3]. Importantly, the patient tolerated the remaining medications initiated upon discharge well, with no recurrence of symptoms.

The mechanism by which metformin induces hemolysis in G6PD-deficient patients remains unclear; however, it is hypothesized to increase oxidative stress on red blood cells (RBCs) due to its toxic accumulation. This differs from the theories proposed by Kashyap et al. and Packer et al. in their case reports, where they suggested an immune-mediated response consistent with either warm autoimmune hemolytic anemia (WAIHA) or drug-induced immune hemolytic anemia (DIIHA), supported by positive direct antiglobulin test (DAT) reactivity in their patients, unlike in our case [8,9].

Metformin-induced hemolysis is considered dose-dependent, with most reported cases indicating that a daily dose of 1000 mg or more can precipitate a crisis [8]. Furthermore, the duration of exposure also plays a role, as most cases occur within two weeks of initiating the drug, consistent with our patient's presentation. Reported cases in adults are limited, with the majority achieving full recovery except for one instance, where the patient had a rapid and fatal course, succumbing within 12 hours of symptom onset, which occurred within two days of starting the drug [8].

Other less common causes of hemolytic anemia such as thrombotic thrombocytopenic purpura (TTP), disseminated intravascular coagulation (DIC), and hemolytic uremic syndrome (HUS) were considered due to the presence of low-grade fever; however, these were ruled out in the absence of other characteristic features such as schistocytes on peripheral blood smear [13]. Hemoglobinopathies were also excluded through normal hemoglobin electrophoresis [14]. Furthermore, a repeat G6PD screen post-discharge confirmed the diagnosis of G6PD deficiency.

Metformin-induced hemolysis is a serious and potentially life-threatening condition, especially in G6PD-deficient patients [8]. The temporal association between starting metformin and the onset of hemolysis was clear in our patient, as symptoms began within two weeks of initiating the medication. The rapid resolution of symptoms following the cessation of metformin (dechallenge) further supports the causality. Although rechallenge was not performed due to ethical concerns, the absence of other potential triggers and the reappearance of symptoms upon re-exposure to the drug in other reported cases provide a strong causal link.

The patient was on multiple medications, but none are known to cause hemolysis in G6PD-deficient patients. This, combined with the rapid resolution of symptoms upon discontinuation of metformin and no recurrence of symptoms on the remaining medications, supports metformin as the causative agent. Causality assessment using the Naranjo Adverse Drug Reaction Probability Scale places this as a probable adverse drug reaction, given the timing, resolution upon drug cessation, and absence of other potential causes [15].

Although our patient had low enzyme levels, some reports indicate that enzyme levels may remain normal during a crisis, yet symptoms resolve upon discontinuing metformin [16]. This suggests that metformin could potentially induce hemolysis in susceptible individuals even without G6PD deficiency. Further studies are necessary to elucidate the exact mechanism and identify potential genetic or biochemical markers that predispose certain individuals to this adverse effect.

## Conclusions

While metformin rarely causes drug-induced hemolysis, it is crucial for physicians to be aware of this potential side effect and consider testing high-risk populations for G6PD enzyme activity before starting metformin treatment. With an increasing number of reported cases, updating the list of medications known to induce hemolysis in G6PD-deficient patients may be warranted.
